# Supplementary material for: Identification of photoperiod-induced specific miRNAs in the adrenal glands of Sunite sheep (Ovis aries)
Source: Front Vet Sci. 2022 Jul 22;9:888207. doi: 10.3389/fvets.2022.888207 (PMC9354845; doi:10.3389/fvets.2022.888207)
Supplement: Supplementary file 1 [file Data_Sheet_1.ZIP › Supplementary file/miRDeep2 file.zip!/miRDeep2 file/Know_Structure/SP42A2/oar-mir-29a.pdf]

miRBase precursor : oar-mir-29a
Total read count : 151569
oar-miR-29a read count : 151542
remaining reads : 27

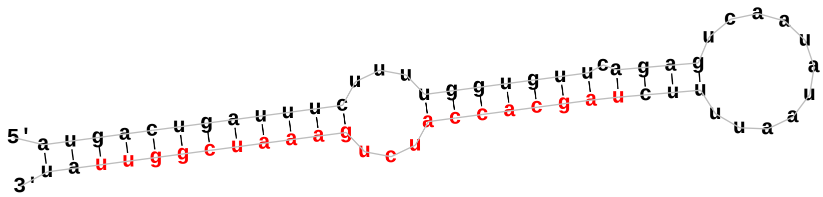

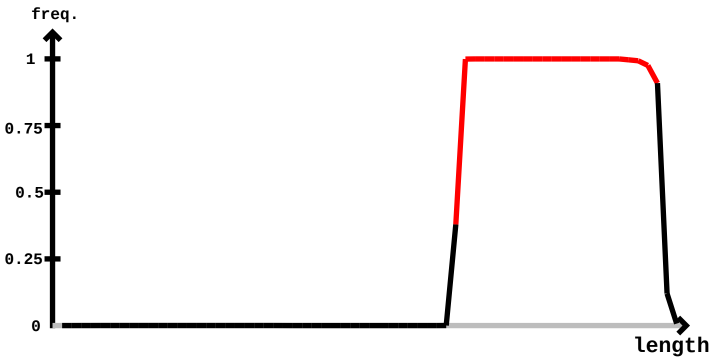

oar-miR-29a

5'- augacugauuucuuuugguguucagagucaauauaauuuucuagcaccaucugaaaucgguuau -3'	exp		
((((((((((((...(((((((.((((...........)))))))))))...))))))))))))	reads	mm	sample
.ugacugauuucuuuugguguu..	3	0	seq
.ugacugauuucuuuugguguuc...	11	0	seq
...acugauuucuuuugguguu..	1	0	seq
...acugauuucuuuugguguuc...	8	0	seq
.....ugauuucuuuugguguuc...	1	0	seq
......................................uucuagcaccaucugaaaucgguu..	2	0	seq
......................................uucuagcaccaucugaaaucgguua.	1	0	seq
....ucuagcaccaucugaaaucgg....	4	0	seq
....ucuagcaccaucugaaaucggu...	13	0	seq
....ucuagcaccauUugaaaucgguu..	2	1	seq
....ucuagcaccaucugaaaucgguu..	7	0	seq
....ucuagcaccaucugaaaucgguua.	2	0	seq
....cuagcaccaucugaaaA.......	1	1	seq
....cuaCcaccaucugaaau.......	1	1	seq
....cuagcaccaucugaaau.......	10	0	seq
....Uuagcaccaucugaaau.......	1	1	seq
....cuagcaccaucugaaauA......	6	1	seq
....cuagcaccaucugaaauc......	101	0	seq
....cuagcaccaucugaaaucg.....	104	0	seq
....cuagcaccauUugaaaucg.....	1	1	seq
....cuagcaccauUugaaaucgg....	2	1	seq
....cuagcaccaucugaaaucgg....	404	0	seq
....cuagcaccaucugaaaucggu...	2666	0	seq
....cuagcaccauUugaaaucggu...	33	1	seq
....cuagcaccauUugaaaucgguu..	188	1	seq
....cuagcaccaucugaaaucgguu..	52828	0	seq
....cuagcaccaucugaaaucgguua.	1035	0	seq
....cuagcaccauUugaaaucgguua.	4	1	seq
....cuagcaccauUugaaaucgguuau	1	1	seq
....cuagcaccaucugaaaucgguuau	80	0	seq
....uagcaccaucugaaauc......	360	0	seq
....uagcaccaucugaaauU......	33	1	seq
....uagcaccaucugaaGuc......	1	1	seq
....uagcUccaucugaaauc......	1	1	seq
....uagcaccaucugaaauA......	10	1	seq

oar-miR-29a

```
augacugauuucuuuugguguucagagucaauauaauuuucuagcaccaucugaaaucgguuau
........................................uagcaccaucugaUauc......    1       1       seq
........................................Aagcaccaucugaaauc......    2       1       seq
........................................uagcaccaucugaaaucg.....    386     0       seq
........................................uagcaccauUugaaaucg.....    45      1       seq
........................................uagcacUaucugaaaucg.....    1       1       seq
........................................uagcaccauUugaaaucgg....    154     1       seq
........................................uagcaccaucugaaaucgg....    2080    0       seq
........................................uagcaccauUugaaaucggu...    868     1       seq
........................................uagcaccaucugaaaucggu...    6462    0       seq
........................................uagcaccaucugaaaucgguu..    63521   0       seq
........................................uagcaccauUugaaaucgguu..    3004    1       seq
........................................uagcaccauUugaaaucgguua.    743     1       seq
........................................uagcaccaucugaaaucgguua.    15268   0       seq
........................................uagcaccauUugaaaucgguuau    57      1       seq
........................................uagcaccaucugaaaucgguuau    965     0       seq
.........................................agcaccaucugaaaucggu...    8       0       seq
.........................................agcaccauUugaaaucggu...    1       1       seq
.........................................agcaccauUugaaaucgguu..    1       1       seq
.........................................agcaccaucugaaaucgguu..    59      0       seq
.........................................agcaccauUugaaaucgguua.    1       1       seq
.........................................agcaccaucugaaaucgguua.    6       0       seq
.........................................agcaccaucugaaaucgguuau    3       0       seq
..........................................gcaccaucugaaaucggu...    1       0       seq
..........................................gcaccaucugaaaucgguu..    5       0       seq
..........................................gcaccaucugaaaucgguuau    1       0       seq
```
